# Emotion control training enhances reappraisal success among individuals with reported ADHD symptoms

**DOI:** 10.1038/s41598-022-18441-z

**Published:** 2022-08-18

**Authors:** Revital Hamerman, Noga Cohen

**Affiliations:** 1 Department of Special Education, University of Haifa, 199 Abba Khoushy Ave, 3498838 Haifa, Israel; 2 Department of Special Education, The Edmond J. Safra Brain Research Center for the Study of Learning Disabilities, University of Haifa, 199 Abba Khoushy Ave, 3498838 Haifa, Israel

**Keywords:** Human behaviour, Emotion

## Abstract

Previous research indicates that training individuals to recruit cognitive control before exposure to negative pictures can facilitate the propensity to use reappraisal and reappraisal success. Individuals with attention deficit hyperactivity disorder (ADHD) experience difficulties in cognitive control and emotion regulation, so they may especially benefit from such training. Individuals reporting high ADHD symptoms and controls were randomly assigned to one of two training conditions. In the high emotion control (H-EC) training condition, negative pictures were typically preceded by a stimulus that recruits cognitive control. In contrast, in the low emotion control (L-EC) training condition, negative pictures were typically preceded by a stimulus that does not recruit cognitive control. Participants were then asked to recall an adverse personal event and to reappraise the event. As predicted, instructed reappraisal was more effective in reducing negative mood in the H-EC training compared to the L-EC training. Furthermore, compared to controls, individuals with reported ADHD symptoms showed a greater propensity to use reappraisal after writing the event and a more considerable reduction in event significance and negativity following the instructed reappraisal assignment. We argue that employing cognitive control over emotional information has a causal role in reappraisal use and success among individuals with ADHD symptoms.

## Introduction

Attention Deficit Hyperactivity Disorder (ADHD) is a neurodevelopmental disorder divided into three subtypes: inattentive, hyperactive-impulsive, and combined type (Diagnostic and Statistical Manual of Mental Disorders; DSM-5; APA^1^). The inattentive subtype is characterized by attention deficits, while difficulties in controlling behavior characterize the hyperactive-impulsive subtype. The combined subtype includes both attention deficits and hyperactive-impulsive symptoms. These three core symptoms of ADHD (inattention, impulsivity, and hyperactivity) may lead to feelings of frustration, poor planning and poor decision-making^2^, dysfunctional interpersonal relationships^3–5^, and impaired academic performance^6^. These, in turn, may impair the ability of these individuals to adapt and cope within the environment^2,7^.

Some of the difficulties individuals with ADHD face may be explained by impairment in emotion regulation^8^. Specifically, compared to controls, individuals with ADHD exhibit a higher prevalence of emotion regulation difficulties, especially in stressful and frustrating situations^9,10^. Among these emotion regulation difficulties is a reduced tendency to use reappraisal^11^, an adaptive emotion regulation strategy in which the individual thinks differently about a situation to reduce negative feelings^12,13^.

Reappraisal is associated with improved emotional health^12,13^ and has been found to moderate the link between stress and inattention symptoms^14^. Namely, while inattention problems are usually associated with increased perceived stress, this link is weaker among individuals who have a greater tendency to engage in reappraisal^7^. Furthermore, less frequent use of reappraisal has been found associated with risk behaviors and psychopathology in ADHD^15,16^. Therefore, it is unfortunate that individuals with ADHD symptoms tend to use reappraisal less frequently than controls^17^, which has been suggested to explain their elevated feelings of distress, anger, guilt, and shame^18^. Nevertheless, when instructed to employ reappraisal, individuals with ADHD symptoms can use this strategy to reduce negative feelings^19^. Furthermore, training individuals with ADHD to employ reappraisal in interventions such as Cognitive Behavioral Therapy (CBT) or Dialectical Behavioral Therapy (DBT) has been found to reduce inattention and impulsivity symptoms in adults^20–26^, as well as in children and adolescents^20,21^.

Recent notions argue that reappraisal may depend on cognitive control^27^. Cognitive control comprises the set of processes that enable individuals to behave according to their goals and ignore irrelevant information. Similarly, reappraisal involves the suppression of irrelevant information to perform goal-oriented behavior^22^. Indeed, several studies found a positive link between cognitive control and reappraisal^23–26,28^. For example, McRae et al.^26^ found that reappraisal is positively associated with specific cognitive control components (working memory capacity and set-shifting costs) for both emotional and neutral material. Similarly, greater use of reappraisal was found to be associated with less emotional interference by aversive images following a flanker task that recruits inhibitory control^29^.

The findings mentioned above show a correlational link between the tendency to use reappraisal and cognitive control. Studies have only recently begun testing whether there is a causal link between cognitive control and the propensity to use reappraisal and between cognitive control and the ability to implement reappraisal when facing a negative event. One way of assessing this causal link is by training individuals with a cognitive control task and measuring whether such training increases the propensity to use reappraisal or the effectiveness of an instructed reappraisal assignment. The results of these studies are inconsistent, with some findings showing that cognitive control training can enhance the use and effectiveness of cognitive reappraisal^24,30–35^, while others fail to find such an effect^36,37^. For example, Cohen and Mor^24^ showed that a training procedure in which participants recruit cognitive control before the presentation of negative pictures enhances reappraisal propensity and the ability of instructed reappraisal to reduce negative mood. In their study, healthy individuals completed a task in which a flanker stimulus was shown before a negative or a neutral picture. Incongruent flanker stimuli consist of a conflict between the target and distractors and are therefore associated with recruitment of cognitive control. Reaction time (RT) to a discrimination task (deciding whether a square is blue or green) at the end of the trial was used to assess the effect of incongruent flankers on emotional interference^38,39^. The authors paired the appearance of incongruent flanker stimuli (that recruit cognitive control) with negative pictures. Therefore, in the training group, participants recruited cognitive control before encountering the emotional information in most (80%) of the trials. In the control group, however, most of the negative pictures were preceded by a congruent flanker stimulus, which does not recruit cognitive control. Following this task, the two groups were asked to recall a personal negative event and reappraise it. The results revealed less emotional interference by negative pictures on reaction times of the discrimination target when these pictures appeared following the recruitment of cognitive control (incongruent flanker stimuli; see also^40,41^). Compared to the control group, the training group reported higher use of reappraisal after recalling the event and greater success in the instructed reappraisal assignment, as indicated by a larger reduction in negative mood. These results are in line with findings showing that transcranial direct current stimulation (tDCS) on brain regions associated with cognitive control decreases arousal ratings in a reappraisal task^42^.

The role of cognitive control in emotion regulation may be especially relevant in the context of ADHD. Individuals with ADHD symptoms show deficits in cognitive control^43–45^, what may explain their emotion regulation difficulties^46,47^. This idea is in line with findings showing that these individuals have difficulties ignoring irrelevant information^43,48^, both neutrally and emotionally valenced^49^. Inability to ignore irrelevant emotional information is indeed associated with reduced ability to use reappraisal^29^ and with elevated levels of depression and rumination^41,50^. Prior studies on ADHD assessed either deficits in cognitive control or deficits in emotion regulation, and therefore it is yet unknown whether cognitive control plays a role in enabling adaptive emotion regulation use among this population.

The current study is the first to examine whether a training procedure in which cognitive control is employed over negatively valenced stimuli can enhance reappraisal ability among individuals reporting high levels of ADHD symptoms. Individuals who reported having ADHD symptoms and controls performed a training task in which cognitive control recruitment (i.e., incongruent flanker stimulus) was paired with negative pictures (high emotion control; H-EC condition) or with neutral pictures (low emotion control; L-EC condition). Reaction time (RT) to a discrimination task (deciding whether a square is blue or green) that appeared at the end of the trial was used to assess the influence of cognitive control on emotional interference^38,39^. This trial sequence, in which a discrimination target is used to assess the interaction between cognitive control and emotional processing is common and was shown to be effective in revealing an interaction between cognitive control and emotion in previous studies^24,40,41,51^. Following the training task, participants were asked to recall a negative personal event and reappraise it^24^. Based on prior literature showing difficulties both in emotion regulation^43,48^ and cognitive control^44,45,52^ among individuals with ADHD symptoms, we expected the high emotion control (H-EC) training would be highly beneficial for these individuals. Specifically, we had several predictions:

### Emotion control training task

- Regarding the flanker task, we expected to replicate the common congruity effect found in this task. Specifically, we expected to find slower RTs for incongruent vs. congruent flanker stimuli. We did not expect this effect to differ between the two training conditions nor between the ADHD symptoms and control groups.
- Considering the emotional interference effect measured using the discrimination task, we expected to replicate the effects observed in Cohen and Mor^24^ showing less emotional interference (slower RTs for discrimination targets that appear after negative vs. neutral pictures) following incongruent vs. congruent flankers. This effect was predicted to be larger in the H-EC condition vs. the L-EC condition. We did not have a specific prediction regarding group difference (controls vs. ADHD symptoms) in this effect.

### Reappraisal assessment task

- Considering reappraisal propensity, we predicted that similar to what was found in Cohen and Mor^24^, participants in the H-EC condition would show a greater propensity to use reappraisal, compared to the L-EC condition. This effect was expected to be larger among individuals reporting high levels of ADHD symptoms compared to the control group.
- Considering reappraisal success, we predicted greater success in implementing the instructed reappraisal assignment in the H-EC vs. the L-EC condition^24^. Specifically, compared to the L-EC group, the H-EC group was expected to report a lower negative mood following reappraising the event vs. before reappraising it. This effect was expected to be larger among individuals reporting high levels of ADHD symptoms compared to the control group.
- An examination of the effects of the instructed reappraisal assignment on the reported negativity and personal significance of the event was conducted on an exploratory basis with no specific predictions.

## Method

### Participants

This study was approved by the institutional review board of the Faculty of Education, University of Haifa (No. 008/19). All methods were carried out in accordance with standard human research ethics guidelines (Declaration of Helsinki) and regulations. Written informed consent was obtained from the participants.

A total of 193 individuals participated in the study (91 controls and 102 with ADHD symptoms). Data of control participants were taken from Cohen and Mor’s^24^ study. All participants had normal or corrected-to-normal vision and were native Hebrew speakers. Participants in the control group reported no history of psychiatric disorders or ADHD, while the group with reported ADHD symptoms included only individuals who reported having ADHD symptoms. We did not conduct a formal ADHD assessment or assessment of comorbid disorders by clinicians. A request for a formal diagnosis was sent to all participants in the ADHD symptoms group who agreed to be contacted by the researchers via email. This request was sent approximately 1.5 years following the end of data collection and 39% of the participants provided official documentation specifying a diagnosis of ADHD. Moreover, in a questionnaire administered to this group at the end of the study, 52% of the participants reported taking medications for ADHD regularly. None of the participants in the control group reported taking a medication for ADHD. Participants in the ADHD symptoms group also filled out a questionnaire assessing ADHD symptoms which indicated that above 80% of the participants reported experiencing a high level of at least five symptoms of inattention, hyperactivity, or impulsivity. Excluding participants who reported lower levels of ADHD symptoms (more than 2SD below the mean score in the ADHD Symptoms Checklist) did not change the pattern of the observed effects.

Participants in the reported ADHD symptoms group were asked to withhold taking medication for their disorder on the day of the experiment. The experiment session was conducted mostly during morning hours meaning that participants were asked to avoid taking their morning pill. Data from 17 participants were removed due to a high error rate in the training task (more than 20% errors in the flanker or the discrimination tasks), and data of two participants were removed because they did not follow the writing task instructions. Additional nine participants were removed as they rated the event negativity/significance/their mood following writing it as 0. Three participants from the ADHD symptoms group were removed as they mentioned taking medicine before the experiment in a questionnaire administered at the end of the study. Therefore, the analyses included 163 participants (see Table 1 for demographic characteristics).

**Table 1:** Descriptive data for sex, age, trait emotion regulation, and ADHD symptoms for the ADHD symptoms and the control group.

	Control		ADHD	
	H-EC	L-EC	H-EC	L-EC
N	39	47	42	35
Sex, % females	79%	55%	43%	46%
Age, mean (SD)	23.33 (1.68)	24.87 (4.26)	24.38 (2.65)	25.14 (3.80)
ERQ-reappraisal	28.18 (7.08)	27.13 (6.39)	27.19 (8.05)	28.83 (7.24)
ERQ-suppression	12.92 (5.21)	11.85 (5.19)	15.05 (4.95)	13.51 (5.87)
ADHD symptoms			45.55 (9.17)	39.40 (12.39)

### Procedure

**Figure 1:**
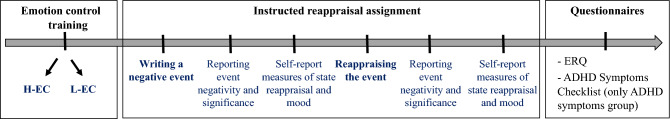
Overall study design. Participants first completed the training task. They were randomly assigned to either the H-EC or the L-EC condition. They then performed the instructed reappraisal assignment. Finally, they completed the Emotion Regulation Questionnaire (ERQ). Participants in the ADHD symptoms group also answered the ADHD Symptoms Checklist.

Participants in both groups were randomly assigned to one of two training conditions: high emotion control (H-EC) and low emotion control (L-EC). The training task was programmed using E-Prime (E-Prime 3 Professional, Psychology Software Tools, Inc., Pittsburgh, PA, USA). After the training task (see below), participants completed an instructed reappraisal assignment that included recalling and writing a negative personal event and reappraising it. Then, participants completed self-report measures of state reappraisal and negative mood. They were also asked about the negativity of the event and its significance. Finally, participants completed the Emotion Regulation Questionnaire^13^ which assesses habitual use of reappraisal and suppression. Participants in the ADHD symptoms group also completed the ADHD Symptoms Checklist which measures ADHD severity based on the DSM-5 (see Fig. 1 for overall study design). Additional questionnaires were administered at the end of the task but are not discussed in the current paper. All data are available at https://osf.io/zpurj/.

#### Emotion control training

**Figure 2:**
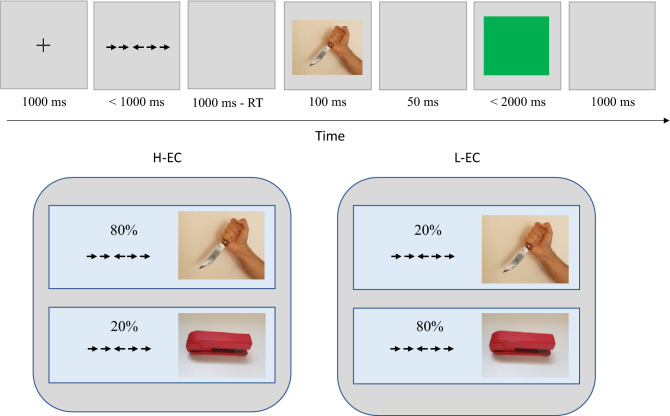
Emotion control training. Participants were required to respond to a flanker stimulus and then to discriminate between a blue and a green square. In the H-EC condition, most (80%) of the negative pictures were preceded by an incongruent flanker stimulus, while in the L-EC condition, most (80%) of the negative pictures were preceded by a congruent flanker stimulus. Illustration images were taken by N.C.

Participants in the two training conditions (H-EC, and L-EC) were asked to respond as rapidly as possible to two tasks on each trial using the computer keyboard^24^. Each trial (see Fig. 2) started with a fixation cross presented for 1000 ms. Next, a flanker stimulus was presented until response, but for no longer than 1000 ms. The flanker stimulus included a line of five arrows. Half of the trials included a congruent stimulus, in which all five arrows point to the same direction (e.g., > > > > >). The other half of the trials included an incongruent stimulus, in which the middle arrow points in the opposite direction of the flankers (e.g., > > < > >). Incongruent flanker stimuli are known to recruit cognitive control due to the conflict between the target arrow and the flankers^53,54^. Following an interval of 1000 ms minus RT to the flanker stimulus, a picture appeared for 100 ms. Half of the trials included a negative picture, while the other half included a neutral picture. Following the picture, a 50 ms interval appeared. Then, a discrimination target (a blue or a green square) appeared until response, but for no longer than 2000 ms. An inter-trial interval (ITI) of 1000 ms ended each trial. Participants were asked to respond to the flanker stimulus by indicating the direction of the middle arrow. Response to the discrimination target included indicating whether the square is blue or green. While the proportions of incongruent and congruent trials, as well as the proportions of negative and neutral pictures, were equal across the H-EC and L-EC conditions, the proportion of the pairing between the flanker stimulus (congruent, incongruent) and the picture valence (negative, neutral) varied between these conditions. Specifically, in the H-EC condition, negative pictures were usually (80%) preceded by incongruent stimuli and seldom by congruent stimuli, while in the L-EC condition, the opposite was true (i.e., 20% of negative pictures were preceded by incongruent stimuli). Therefore, participants in the H-EC condition recruited cognitive control before most of the negative stimuli. The task consisted of 320 randomly presented trials. Picture stimuli included 12 negative and 12 neutral pictures selected from the International Affective Pictures System (IAPS^55^; for more details, see^24^). Sixteen practice trials were given before the actual task.

#### Instructed reappraisal assignment

The instructed reappraisal assignment consisted of two stages: recalling a negative personal event and reappraising the event^24^. Participants were given four minutes to recall an event that made them feel bad about themselves and write about it. Then they were required to indicate the extent to which the event was negative (“How negative is the event for you*?*) and significant (“How significance is the event for you?”) for them. Afterward, they completed state measures of reappraisal and mood (see below). For all measures, participants answered by using a mouse cursor to indicate a location on a visual analog scale that ranged from highly agree (0) to highly disagree (100). Subsequently, participants performed an instructed reappraisal task in which they were asked to reinterpret the event to make it seem less negative. They were given four minutes to write how they would reappraise the event. Following the instructed reappraisal assignment, participants again answered the questions about negativity and significance and completed the state measures.

#### Self-report state measures of reappraisal and mood

State reappraisal was assessed using five statements modified from the ERQ^13^. The assessment of negative mood included four items (e.g.,” right now, I feel sad”). For more details, see Cohen and Mor^24^. Participants answered by clicking with a mouse cursor on a visual analog scale (VAS) ranging from 0 (not at all) to 100 (very much).

### Trait questionnaires

#### ADHD symptoms checklist

As noted above, this study did not conduct a formal ADHD assessment by clinicians. It relied on reported ADHD symptoms. As participants in the control group reported having no psychiatric disorders including ADHD, the level of ADHD symptoms was assessed only among individuals recruited for the ADHD symptoms group. This assessment was conducted using the DSM-5 ADHD Symptom Checklist. This questionnaire includes 18 statements modified from the Diagnostic and Statistical Manual of Mental Disorders (DSM-5; APA^1^). The questionnaire included nine items assessing inattention difficulties and nine items assessing hyperactivity/impulsivity. Participants answered on a scale ranging from 1 (never) to 7 (often). The questionnaire is available at https://osf.io/zpurj/.

#### Emotion regulation questionnaire (ERQ^13^)

The ERQ includes 6 statements measuring habitual reappraisal (e.g., "When I want to feel more positive emotion (such as joy or amusement), I change what I'm thinking about”) and 4 statements measuring suppression (e.g., “I control my emotions by not expressing them”). The scale ranges from 1—strongly disagree to 7—strongly agree.

## Results

### Training task

#### Data reduction

Trials with incorrect responses on the flanker and discrimination tasks were eliminated from the analysis (4.89% and 3.69% respectively in the ADHD symptoms group; 4.39% and 4.42% respectively in the control group). Furthermore, we excluded trials with extreme RTs (2.5 SD above the participant’s mean RT), resulting in the exclusion of 2.04% trials in the flanker task and 1.68% trials in the discrimination tasks for the ADHD group, and 2.42% trials in the flanker task and 2.50% trials in the discrimination tasks for the control group.

#### Flanker task

RTs in the flanker task were subjected to a two-way mixed analysis of variance (ANOVA), with congruity (congruent vs. incongruent) as a within-subjects factor and condition (H-EC, L-EC) and group (ADHD symptoms, control) as between-subject factors. A typical main effect of congruity was found, *F*(1, 159) = 1347, *p* < 0.001, partial η^2^ = 0.89, reflecting faster RTs on congruent trials (M = 480 ms, SD = 50) than on incongruent ones (M = 544 ms, SD = 51). The interaction between congruity and training condition was not significant, *F*(1, 159) = 0.71, *p* = 0.40, partial η^2^ = 0.004, indicating that the training conditions did not differ in congruity. Moreover, no interaction was found between congruity and group, *F*(1, 159) = 1.37, *p* = 0.24, partial η^2^ = 0.009, or between congruity, condition and group, *F*(1, 159) = 0.61 *p* = 0.44, partial η^2^ = 0.004.

#### Emotional interference

RTs to the discrimination targets were subjected to a three-way mixed ANOVA. Flanker congruity (congruent vs. incongruent) and picture valence (negative vs. neutral) served as within-subjects factors, and condition (H-EC vs. L-EC) and group (ADHD symptoms vs. control) served as between-subjects factors. A main effect of congruity was found indicating slower RTs for discrimination targets that appeared after incongruent vs. congruent stimuli, *F*(1, 159) = 5.27, *p* = 0.02, partial η^2^ = 0.03. As predicted, the interaction between congruity, valence, and condition was significant, *F*(1, 159) = 5.03, *p* = 0.03, partial η^2^ = 0.03. All other interactions with experiment or group were not significant. Post hoc analysis revealed that the interaction between congruity and valence was not significant in the L-EC condition *F*(1, 81) = 1.88, *p* = 0.17, partial η^2^ = 0.02, in which a main effect for valence was observed, *F*(1, 81) = 6.42, *p* = 0.02, partial η^2^ = 0.07, with overall slower responses following negative compared to neutral pictures. In contrast, the congruity–valence interaction was marginally significant in the H-EC condition, *F*(1, 80) = 3.28, *p* = 0.07, partial η^2^ = 0.04. In this training condition, the response to discrimination targets was delayed following negative (M = 569, SD = 81) compared to neutral (M = 556, SD = 78) pictures when a congruent stimulus preceded the picture. This effect was smaller when an incongruent stimulus preceded the picture (Mean RT following negative pictures: M = 570, SD = 85; Mean RT following neutral pictures: M = 565, SD = 84).

### Reappraisal assessment task

#### Training effect on the propensity to use reappraisal

To examine the hypothesis that participants in the H-EC condition would demonstrate a higher propensity to use reappraisal than those in the L-EC condition, reappraisal scores were subjected to a two-way ANOVA. Training condition (H-EC vs. L-EC) and group (ADHD symptoms vs. control) were used as between-subject factors, and state reappraisal after writing up the event served as the dependent variable. Contrary to our prediction, the effect of condition was not significant, *F*(1, 155) = 1.94, *p* = 0.17, partial η^2^ = . 01, indicating that individuals in the H-EC training condition did not report using reappraisal more than individuals in the L-EC condition. However, the effect of group was significant, *F*(1, 155) = 10.87, *p* < 0.001, partial η^2^ = 0.07, indicating that individuals with ADHD symptoms reported greater use of reappraisal than controls. The interaction between condition and group was not significant, *F*(1, 155) = 2.40, *p* = 0.12, partial η^2^ = 0.02.

#### Training effect on reappraisal success

**Figure 3:**
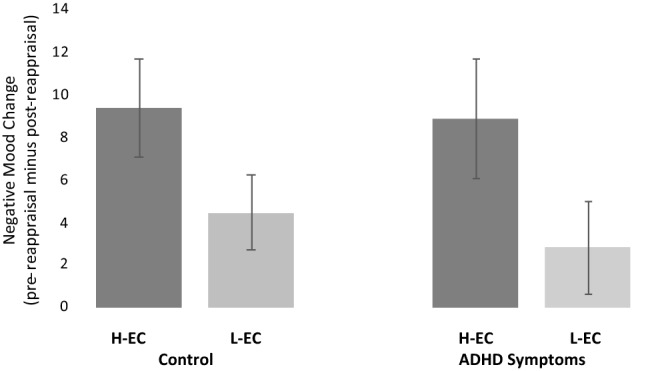
Comparison between the control and ADHD symptoms groups in the two training conditions (H-EC vs. L-EC). The figure shows participants’ self-reported change in negative mood. Change scores = ratings after writing the event (pre-reappraisal) minus ratings after the instructed reappraisal assignment (post-reappraisal). Higher positive values indicate a larger reduction in negative mood. Error bars represent standard errors.

To examine the hypothesis that participants in the H-EC condition would demonstrate a higher degree of reappraisal success than those in the L-EC condition (as indicated by a greater reduction in negative mood following the instructed reappraisal), mood scores were subjected to a three-way mixed ANOVA. Time (pre-reappraisal vs. post-reappraisal) served as a within-subjects factor, while condition (H-EC vs. L-EC) and group (ADHD symptoms vs. control) served as between-subjects factors. A main effect of time was found, *F*(1, 155) = 30.49, *p* < 0.001, partial η^2^ = 0.16, reflecting reduction in negative mood following the instructed reappraisal assignment. The interaction between time and condition was also significant, *F*(1, 155) = 5.70, *p* = 0.02, partial η^2^ = 0.04, indicating a larger reduction in negative mood following the instructed reappraisal assignment in the H-EC training condition than in the L-EC condition. No interaction was found between time and group, *F*(1, 155) = 0.22, *p* = 0.64, partial η^2^ = 0.001, or between time, condition and group, *F*(1, 155) = 0.08, *p* = 0.81 partial η^2^ < 0.001 (see Fig. 3).

#### The link between the propensity to use reappraisal and reappraisal success

A correlation analysis was conducted to assess whether the level of reported spontaneous reappraisal following the event writing was correlated with reappraisal success (change in negative mood from after writing the event to after reappraising it). When looking at the entire sample, this correlation was not significant (*r* = − 40.13, *p* = 0.10). To further test whether the interaction between training group (H-EC, L-EC) and the propensity to use reappraisal predicted reappraisal success, a hierarchical regression model was conducted. Negative mood difference scores served as the dependent variable. Training condition (H-EC, L-EC) and reappraisal propensity scores were entered first as independent variables into the model, followed by the interaction between these two variables. Results showed that, in the first model, only training condition significantly predicted the difference in negative mood (β = − 0.17, *t* =  − 2.17, *p* = 0.03). This model accounted for 4.6% of the variance in negative mood change, *F*(2, 156) = 3.73, *p* = 0.03. The interaction between training condition and spontaneous reappraisal, entered in the second step, did not significantly predict change in negative mood (β =  − 0.00, *t* =  − 0.003, *p* = 0.998). The second model accounted for 4.6% of the variance, *F*(3, 155) = 2.47, *p* = 0.06, and did not add significantly to the first model, *F*_change_(1, 155) = 0.00, *p* = 0.998. Together, these findings indicate no link between reappraisal propensity and reappraisal success.

#### Training effect on self-reported negativity of the event

Negativity scores were subjected to a three-way mixed ANOVA. Time (pre-reappraisal vs. post-reappraisal) served as a within-subjects factor and condition (H-EC vs. L-EC), and group (ADHD symptoms vs. control) served as between-subjects factors. A main effect of time was found, *F*(1, 155) = 147.99, *p* < 0.001, partial η^2^ = 0.49, reflecting a reduction in the reported negativity of the event following the instructed reappraisal assignment. There was no interaction between time and condition, *F*(1, 155) = 0.61, *p* = 0.44, partial η^2^ = 0.004, but the interaction between time and group was significant, *F*(1, 155) = 9.27, *p* = 0.003, partial η^2^ = 0.06, indicating a larger reduction in reported negativity following the instructed reappraisal assignment among individuals with reported ADHD symptoms compared to controls. There was no interaction between time, condition and group, *F*(1, 155) = 0.15, *p* = 0.70, partial η^2^ = 0.001.

#### Training effect on the self-reported significance of the event

**Figure 4:**
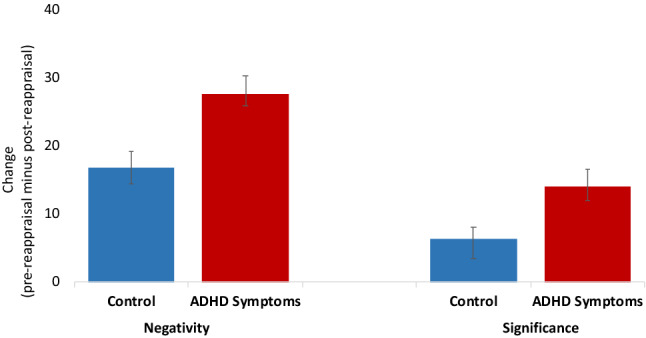
Comparison between the control and ADHD symptoms groups in the self-reported change in negativity and significance ratings. Change scores = ratings after writing the event (pre-reappraisal) minus ratings after the instructed reappraisal assignment (post-reappraisal). Higher positive values indicate a larger reduction in the negativity and significance ratings. Error bars represent standard errors.

Significance scores were subjected to a three-way mixed ANOVA. Time (pre-reappraisal vs. post-reappraisal) served as a within-subjects factor, and condition (H-EC vs. L-EC) and group (ADHD symptoms vs. control) served as between-subjects factors. A main effect of time was found, *F*(1, 155) = 46.50, *p* < 0.001, partial η^2^ = 0.23, reflecting a reduction in reported significance following the instructed reappraisal assignment. The interaction between time and condition was not significant, *F*(1, 155) = 2.94, *p* = 0.09, partial η^2^ = 0.02. However, the interaction between time and group was significant, *F*(1, 155) = 6.11, *p* = 0.02, partial η^2^ = 0.04, indicating a larger reduction in significance following the instructed reappraisal assignment among individuals with reported ADHD symptoms than among controls. There was no interaction between time, condition and group, *F*(1, 155) = 0.50, *p* = 0.48, partial η^2^ = 0.003 (see Fig. 4).

## Discussion

The current study examined whether training individuals reporting high levels of ADHD symptoms to employ cognitive control over negative information improves their emotion regulation ability in terms of their propensity to use reappraisal and the success of instructed reappraisal. To that end, we compared individuals with reported ADHD symptoms to controls. The results revealed no difference between the ADHD symptoms and control groups in cognitive control (congruity effect) or emotion control (interaction between flanker type and valence). Furthermore, training effect on reappraisal success was similar among both groups, implying that individuals with reported ADHD symptoms can benefit from such training similarly to controls. Nevertheless, regardless of the training condition, individuals reporting ADHD symptoms showed a higher propensity to use reappraisal than controls following the event writing and demonstrated a more considerable reduction in event significance and negativity following the instructed reappraisal assignment.

The current study's findings revealed that when recalling a negative event, individuals reporting ADHD symptoms show a higher propensity to use reappraisal than controls. Furthermore, the reappraisal assignment was more successful in reducing the reported negativity and significance of the event among these individuals. Specifically, while the negativity and significance of the event were reduced in both samples following the instructed reappraisal assignment, this effect was more prominent among individuals reporting ADHD symptoms. These results support prior findings showing that individuals with ADHD symptoms demonstrate reappraisal success similar to that of controls^56^ and findings showing that these individuals tend to use positive reappraisal in stressful situations^57^, especially when they are instructed to do so^19^. Therefore, these results imply that while individuals with ADHD symptoms may show lower levels of trait reappraisal^11,17,58^, they do use reappraisal when recalling an adverse event, as well as able to use reappraisal effectively to reduce negative mood, even more than typical individuals. The ability of individuals with ADHD symptoms to reappraise is critical as reappraisal has been shown to moderate the relationship between inattention and perceived stress^7^. Furthermore, the efficacy of instructed reappraisal among this population is especially promising as it suggests that they can be taught to implement reappraisal, what may lower their tendency to use maladaptive coping strategies^56^.

Contrary to previous findings showing that individuals with ADHD exhibit difficulties in cognitive control tasks that require inhibitory control^44,45,59,60^, our results did not reveal any difference in cognitive control ability (congruity effect) between individuals reporting ADHD symptoms and controls. Although numerous prior studies indicated cognitive control difficulties among these individuals^61^, some failed to find a difference between ADHD and typical individuals in cognitive control tasks^62^.

Our findings also do not support prior findings showing increased emotional interference or emotion control difficulties (altered interaction between congruity and valence) among individuals with ADHD symptoms^43,48^. Prior research indicates that adults with ADHD show increased emotional responsivity toward negative stimuli^17^, and exhibit greater cognitive effort when employing emotion regulation than controls^17^. These findings are supported by brain imaging studies that indicate emotional hyperresponsivity in adults with ADHD compared to controls^56^. These studies, however, did not assess the direct influence of cognitive control on emotion, which seems to be intact in these individuals, according to our findings.

Regarding training effect on reappraisal propensity and success, while we did not replicate the findings of Cohen and Mor^24^ showing that H-EC training is associated with a higher propensity to use reappraisal, we did replicate the findings showing a greater success in instructed reappraisal in the H-EC compared to the L-EC condition. This effect was not dependent on the group (ADHD symptoms, control) and therefore implies that individuals with reported ADHD symptoms could benefit from the training similarly to controls. These results are encouraging as it seems that individuals with ADHD symptoms can use reappraisal effectively when instructed to do so. Furthermore, our results show that similarly to typical individuals, those with reported ADHD symptoms show enhanced reappraisal success following a training procedure in which cognitive control is paired with negatively-valenced stimuli (high emotion control training). Therefore, although individuals with ADHD symptoms were found to be characterized by both cognitive control and emotion regulation difficulties, they seem to benefit from both emotion control training and instructed reappraisal assignment.

The current study has several limitations. First, participants were recruited based on their self-report of having or not having ADHD. Although we did not perform a formal diagnosis of ADHD and other comorbid disorders by clinicians, we were able to get official documentation from almost 40% of the participants in the ADHD symptoms group regarding their ADHD diagnosis. Furthermore, more than half of the participants in this group indicated taking medications for ADHD, and above 80% reported high levels of inattention, hyperactivity, and impulsive symptoms in the DSM-5 ADHD Checklist questionnaire. The data of the control group was taken from a previous study^24^ that was not aimed at examining ADHD-related effects and therefore we did not administer the ADHD Checklist questionnaire. However, the control group included only participants reporting no current or past ADHD diagnosis. We also asked participants in this group about medication use and none of them reported taking medication for ADHD. However, future studies should conduct a formal diagnosis to assess ADHD in both groups. A second limitation of the current study is that no pre-training assessment of spontaneous reappraisal and reappraisal success was administered. The current study aimed at examining the influence of emotion control training on a relatively ecological measure of reappraisal that consisted of the writing of a recent personal event. Participants may find it difficult to find two upsetting events that happened recently, and these events may differ in their emotional impact, with the first one probably being more negative than the second one. Therefore, the reappraisal assignment was administered only following the training (for similar designs see^24,41^). To assess training-related changes in reappraisal propensity and effectiveness future studies may examine both pre- and post-training reappraisal success by using tasks that do not involve recalling a personal event. These tasks can include, for example, reappraising emotional images or unpleasant movie clips. It is noteworthy that trait reappraisal levels, measured using the ERQ, did not differ between the ADHD symptoms and control groups in our study (see also Bodalski et al.,^58^), indicating no baseline difference in the tendency to use reappraisal. Trait suppression level, however, was higher for the ADHD symptoms vs. the control group, similar to what was found in other studies (e.g., Materna et al. ^56^). A third limitation is that an unequal number of women and men participated in the two groups (ADHD symptoms—45% women, controls—64% women). This difference is reasonable considering that ADHD is more prevalent among men than among women. To ensure that the effects were not related to gender, we did control for gender in our analyses and found no main effect for gender or interaction between gender and any of the other dependent variables.

All in all, this study suggests that individuals with reported ADHD symptoms could benefit from a training procedure that pairs cognitive control with negative information. Specifically, emotion control training was found to enhance the effectiveness of instructed reappraisal among this population, similarly to what was found among controls. Moreover, individuals with reported ADHD symptoms showed a more considerable benefit for the instructed reappraisal assignment than controls, indicating their ability to use reappraisal effectively when instructed to do so. We hope that the current research will lead to further testing and ultimately to the development of effective interventions for individuals with ADHD symptoms.

## Data Availability

The datasets generated and/or analyzed during the current study are available in the Open Science Framework (OSF) repository, https://osf.io/zpurj/.
